# Characterization of Human Vaginal Mucosa Cells for Autologous In Vitro Cultured Vaginal Tissue Transplantation in Patients with MRKH Syndrome

**DOI:** 10.1155/2014/201518

**Published:** 2014-08-05

**Authors:** Cristina Nodale, Enrica Vescarelli, Sirio D'Amici, Diana Maffucci, Simona Ceccarelli, Marco Monti, Pierluigi Benedetti Panici, Ferdinando Romano, Antonio Angeloni, Cinzia Marchese

**Affiliations:** ^1^Department of Experimental Medicine, Sapienza University of Rome, 00161 Rome, Italy; ^2^Department of Gynecology and Obstetrics, Sapienza University of Rome, 00161 Rome, Italy; ^3^Department of Public Health and Infectious Diseases, Sapienza University of Rome, 00161 Rome, Italy; ^4^Department of Molecular Medicine, Sapienza University of Rome, 00185 Rome, Italy

## Abstract

Mayer-Rokitansky-Küster-Hauser (MRKH) is a rare syndrome characterized by congenital aplasia of the uterus and vagina. The most common procedure used for surgical reconstruction of the neovagina is the McIndoe vaginoplasty, which consists in creation of a vaginal canal covered with a full-thickness skin graft. Here we characterized the autologous in vitro cultured vaginal tissue proposed as alternative material in our developed modified McIndoe vaginoplasty in order to underlie its importance in autologous total vaginal replacement. To this aim human vaginal mucosa cells (HVMs) were isolated from vaginal mucosa of patients affected by MRKH syndrome and characterized with respect to growth kinetics, morphology, PAS staining, and expression of specific epithelial markers by immunofluorescence, Western blot, and qRT-PCR analyses. The presence of specific epithelial markers along with the morphology and the presence of mucified cells demonstrated the epithelial nature of HMVs, important for an efficient epithelialization of the neovagina walls and for creating a functional vaginal cavity. Moreover, these cells presented characteristics of effective proliferation as demonstrated by growth kinetics assay. Therefore, the autologous in vitro cultured vaginal tissue might represent a highly promising and valid material for McIndoe vaginoplasty.

## 1. Introduction

Mayer-Rokitansky-Küster-Hauser syndrome (MRKH) is a rare disease occurring in 1 out of 4500 female births. It is characterized by congenital aplasia of the uterus and the upper two-thirds of the vagina in women with a normal female karyotype and secondary sexual characteristics [1], and it may occur as isolated (type I) or associated with renal, skeletal, hearing, and cardiac malformations (type II) [2]. The first clinical symptom is primary amenorrhea. At the present the aetiology of this syndrome is still unknown and MRKH is considered a multifactorial disorder.

Several techniques, both surgical and nonsurgical, are available for creation of the neovagina aiming to allow normal sexual life. Among the surgical approaches, McIndoe vaginoplasty is the most widely performed. The procedure consists in the creation of a vaginal tunnel that is subsequently covered by a full-thickness skin graft [3]. In the modified McIndoe technique other materials are used to line the neovaginal cavity, such as autologous human amniotic membranes [4], peritoneal layers of the pouch of Douglas [5], and intestinal grafts [6]. In 2007, we presented the first case of autologous in vitro cultured vaginal tissue transplantation for the epithelialization of the neovagina walls [7].

In this study we further characterized the cell cultures established in our laboratory from vaginal biopsies and used for the autologous in vitro cultured vaginal tissue transplantation. Our aim was to demonstrate the epithelial origin of the obtained human vaginal mucosa cell cultures, highlighting their importance as material of choice in the modified McIndoe vaginoplasty.

## 2. Methods

### 2.1. Ethics Statements

The use of clinical samples (vaginal biopsies) for cell culture characterization complied with the Declaration of Helsinki 1975, revised in 2008, and has been approved by the Institutional Review Board of Department of Obstetrics-Gynecology and Urology of the Sapienza University of Rome. Written consent was obtained from all subjects before inclusion in the study.

### 2.2. Patients

Vaginal biopsies were obtained from nine women aged 16–32 years (mean age 22.2 ± 6.2) who presented primary amenorrhea due to uterovaginal aplasia, diagnosed by clinical examination, transabdominal and pelvic ultrasonography, nuclear magnetic resonance (NMR), and/or vaginoscopy. All patients had a normal 46, XX karyotype. The characteristics of patients are reported in Table 1.

### 2.3. Cell Cultures

Primary cultures of human vaginal mucosa cells (HVMs) were established from 1 cm^2^ full-thickness mucosal biopsy of the vaginal vestibule of MRKH patients. Following enzymatic dissociation, cells were seeded onto collagen IV (10 mg/mL) coated culture plates and maintained in serum-free, estrogen-free Keratinocyte Growth Medium (KGM; Lonza Milano S.r.l., Milan, Italy), with medium change twice a week. MCF-7 cells were obtained from the American Type Culture Collection (ATCC-LGC Promochem, Teddington, UK) and cultured in Dulbecco's Modified Eagle's Medium (DMEM; Invitrogen, Karlsruhe, Germany), supplemented with 10% fetal bovine serum (FBS; Invitrogen) and antibiotics. Primary cultures of human fibroblasts (HF) were established from 1 cm^2^ full-thickness skin biopsy from a healthy donor, as previously described [8] and maintained in DMEM containing 10% FBS. The morphology was evaluated with a phase contrast microscopy.

### 2.4. Growth Kinetics and Viability

HVMs obtained from vaginal mucosal biopsy of MRKH patients were seeded onto 24-well plates coated with collagen IV, at a density of 1 × 10^4^ cells/well. At several time points (days 3, 6, 9, 12, and 15) after seeding, cell number and viability were determined in triplicate wells by trypan blue exclusion assay (Sigma-Aldrich, Milan, Italy) and using a hemocytometer. The viable cells were unstained and counted on the basis of their trypan blue exclusion, while dead cells were stained blue.

### 2.5. Periodic Acid-Schiff Staining

HVMs were stained with Periodic acid-Schiff (PAS) staining and counterstained with hematoxylin to evaluate glycogen deposits. Briefly, cells were fixed in 4% formaldehyde in PBS for 30 sec, washed with PBS and then with distilled water, stained for 5 min with periodic acid, and washed with distilled water. Cells were then stained with Schiff's reagent for 15 min, washed with water for 5 min, counterstained for 1 min with hematoxylin solution, and extensively washed with water for 5 min before microscopic examination and imaging.

### 2.6. Immunofluorescence

Cells, grown on coverslips, were processed for immunofluorescence analysis as previously described [9] and incubated with cytokeratin 19 (K19) antibody (1 : 100 in PBS; Santa Cruz Biotechnology, Santa Cruz CA, USA). Primary antibody was visualized using the appropriate FITC-conjugated IgG (1 : 100 in PBS; Jackson ImmunoResearch Laboratories, West Grove, PA, USA).

### 2.7. RNA Preparation

Total RNA from cultures derived from vaginal mucosa was extracted using TRIzol reagent (Invitrogen, Milan, Italy) following the manufacturer's instructions. RNA samples were quantified using a NanoDrop ND-1000 spectrophotometer (NanoDrop, Wilmington, DE, USA) and evaluated for degradation using an Agilent 2100 Bioanalyzer (Agilent Technologies, Santa Clara, CA, USA).

### 2.8. Quantitative Real-Time PCR (qRT-PCR)

1 *μ*g of total RNA was reverse transcribed using High Capacity RNA to cDNA Kit (Applied Biosystems by Life Technologies, Carlsbad, CA, USA) according to the manufacturer's instructions. cDNA was diluted 1 : 5 and then used for amplification of K5, KGFR, and vimentin using the appropriate TaqMan gene expression assay kits (Applied Biosystems). A total of 2 *μ*L/well of template was added to the sample wells along with Taqman Universal PCR master mix at a concentration of 1x and water to a volume of 25 *μ*L/well. Assays were conducted in triplicate on an ABI 7500 Real-Time instrument (Applied Biosystems) using the following conditions: 50°C for 2 min, 95°C for 10 min, and then 95°C for 15 sec and 60°C for 1 min, repeated 40 times. GAPDH mRNA was used as an endogenous control and Ct value was used as an indicator for the expression of the target.

### 2.9. Western Blot Analysis

HVMs, MCF-7, and HF were lysed in RIPA buffer. Total proteins (50 *μ*g) were resolved under reducing conditions by 8–15% SDS-PAGE and transferred to Immobilon-FL membranes (Millipore). Membranes were incubated overnight at 4°C with K19 (1 : 200 dilution; Santa Cruz Biotechnology), SC-101 to detect KGFR (1 : 1000 dilution) [10], or with vimentin (1 : 1000 dilution; Stemgent, CA, USA) followed by goat anti-mouse or goat anti-rabbit horseradish peroxidase (HRP)-conjugated secondary antibody (Sigma-Aldrich). Bound antibody was detected by enhanced chemiluminescence detection reagents (Pierce Biotechnology Inc., Rockford, IL, USA) according to manufacturer's instructions.

## 3. Results

### 3.1. Growth Kinetics of HVMs Cultures

**Figure 1:**
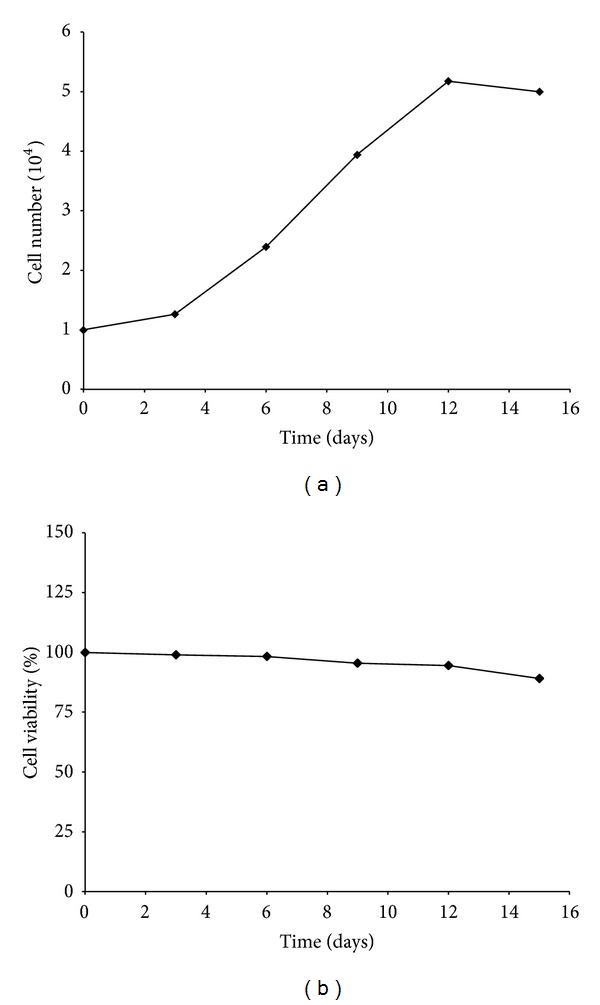
Growth kinetics and viability of HVMs. Live cell number (a) and viability (b) of cultured HVMs harvested from MRKH patients were measured every three days of culture. Cell viability was expressed as percentage of cells viable after 3–6–9–12 days of culture (day 0, 100% living cells). Reported values are the mean of three replicates.

We performed a growth kinetics assay to assess the proliferation capabilities of the HVMs primary cultures. Cells were counted at subsequent time intervals (days 3, 6, 9, 12, and 15) after seeding and their number was plotted against the time points, showing an initial lag phase and an exponential log phase followed by a stationary phase (Figure 1(a)). After the initial lag phase in which cells grew slowly, there was a steady increase in the slope of the growth curve from day 3 to day 12. By the 12th day, HVMs reached the stationary phase due to confluence. During this phase we obtained the fully differentiated mucosal tissue that is used for autologous in vitro cultured vaginal tissue transplantation.

Regarding the cell survival, we found that the percentage of viable HVMs still remained 95% after 12 days of culture and slightly decreased as the cells reached confluence (Figure 1(b)).

These results indicated that HVMs obtained from vaginal mucosal biopsy of MRKH patients can be expanded in culture to form epithelial confluent layers.

### 3.2. Morphology and Expression of K19 in HVMs

**Figure 2:**
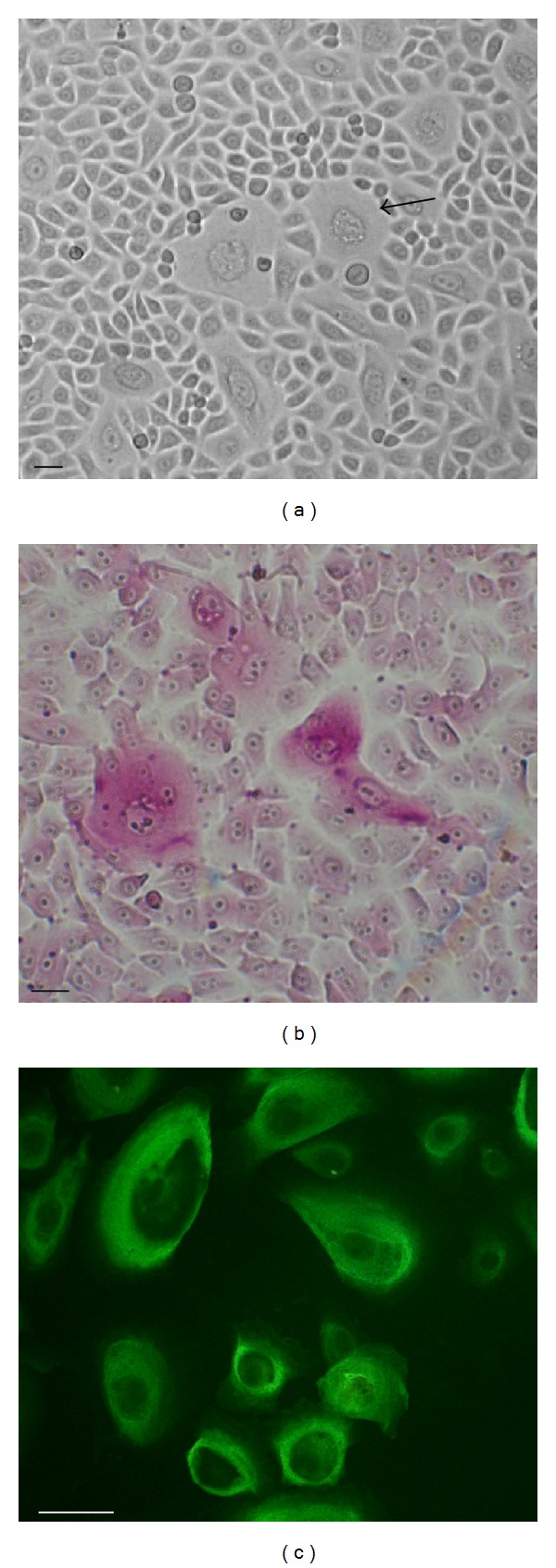
Characterization of cultured vaginal mucosa cells (HVMs). (a) Representative phase contrast image of cell cultures morphology. Muciparous cell is indicated by arrow. Scale bar, 100 *μ*m. (b) Representative PAS staining of HMVs cultures showing PAS positive cells. Scale bar, 100 *μ*m. (c) Expression of K19 in HVMs by immunofluorescence. Image is representative of three independent samples. Scale bar, 100 *μ*m.

The morphology of HMVs derived from MRKH patients was assessed by phase contrast microscopy. The isolated cells exhibited a cobble-stone-like shape, were tightly connected, and had a clear boundary (Figure 2(a)). Mucified cells are scattered among these epithelial cells.

We also analyzed the ability of HVMs to produce mucus, using PAS, which stains structures, such as the mucus, containing a high proportion of carbohydrate macromolecules and epithelial mucins. Microscopy analysis revealed the presence of mucified cells in HMVs population, characterized by the presence of secretory vesicles and positive PAS staining (pink) (Figure 2(b)).

To confirm the epithelial origin of HVMs, we also assessed the expression of K19, an important epithelial marker, by immunofluorescence. Consistent with the morphology, K19 was detected in all HVMs cultures analyzed, thus confirming their epithelial nature (Figure 2(c)).

### 3.3. Expression of Epithelial and Mesenchymal Markers by Western Blot and qRT-PCR Analyses

**Figure 3:**
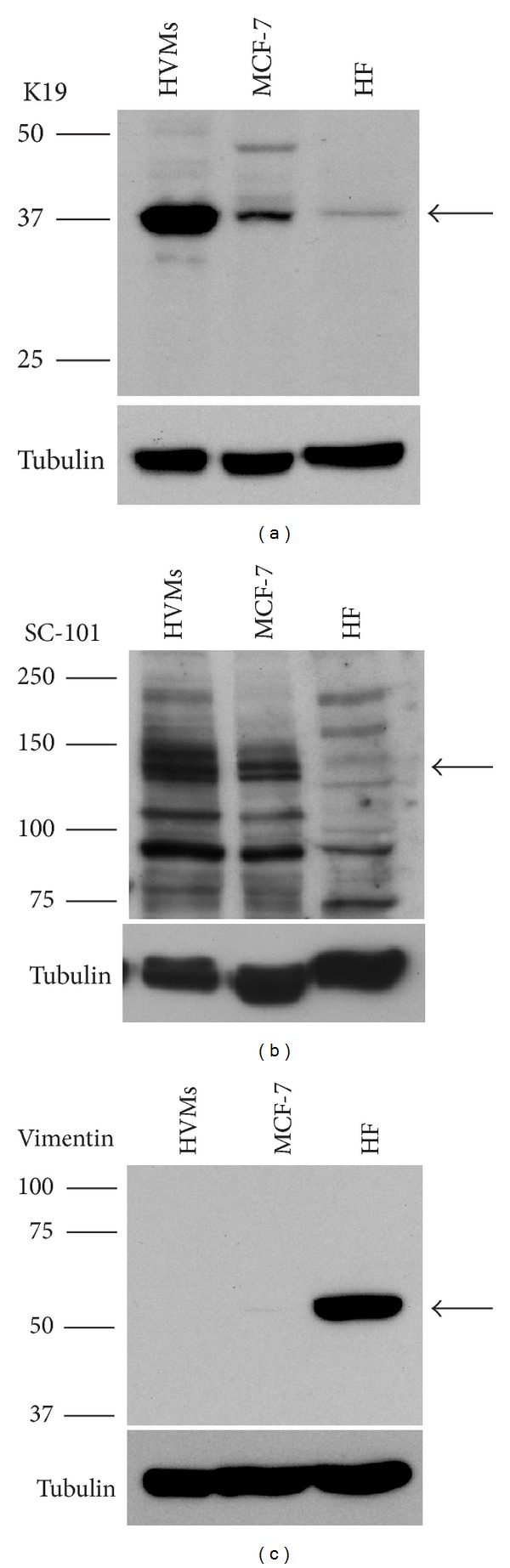
Expression of epithelial and mesenchymal markers in HVMs assessed by Western blot analysis. Western blot analysis of K19 (40 kDa, arrow) (a), SC-101 (145 kDa, arrow) (b), and vimentin (57 kDa, arrow) (c) in HVMs whole cell lysates. MCF-7 and HF were used as positive or negative control. Western blot with anti-tubulin antibody was used as loading control. The images are representative of at least three independent experiments.

We performed Western blot analysis in order to verify the expression of the epithelial markers K19 and keratinocyte growth factor receptor (KGFR) (Figure 3). Moreover, we evaluated the absence of expression of vimentin, a mesenchymal marker, to confirm the real epithelial origin of HVMs (Figure 3). We used MCF-7 and HF cell lines as positive or negative control.

Both K19 (Figure 3(a)) and KGFR (Figure 3(b)) were detected in HVMs and in MCF-7, but not in HF. Furthermore the epithelial origin was confirmed by no detection of vimentin in HVMs and MCF-7 lysates (Figure 3(c)).

We next investigated mRNA expression of another specific epithelial marker, the cytokeratin 5 (K5), along with KGFR gene. Vimentin was used to confirm no mesenchymal origin of HVMs cells by qRT-PCR. The presence of K5 and KGFR was confirmed by the low Ct value, while vimentin was barely detectable (Table 2). The expression of the two epithelial markers and the absence of vimentin are directly consistent with the epithelial cell lineage of HVMs.

Thus, both qRT-PCR and Western blot analysis results confirmed the epithelial origin of vaginal cell cultures.

## 4. Discussion

The McIndoe technique is considered a valid treatment option for vaginoplasty, but no consensus has been reached on what lining material should be used. Amniotic membranes, inert materials, and oral mucosa have all been used to improve the short- and long-term results. In 2007 we have reported for the first time the use of autologous in vitro cultured vaginal tissue for the neovagina canal lining [7]. Recently, another study presented the use of autologous epithelial and muscle cells isolated from vulvar biopsies to reconstruct engineered vaginal organs using a biodegradable scaffold obtained from xenogeneic intestinal decellularised submucosa [11]. However, this pilot study presented considerable limitations, due to the small number of patients and to some issues concerning the procedure, such as the higher number of passages for primary cultures, the higher costs of the procedure due to the need of a scaffold, and the potential risks related to the use of an organoid tissue that, although investigated, still need a deeper evaluation of safety and efficacy.

In our study, we strongly suggest the use of autologous cultured vaginal tissue as material of choice in modified McIndoe vaginoplasty, since safety and long-term results of this cell based therapy have been widely demonstrated. Moreover, our characterization of the in vitro reconstructed vaginal mucosa further confirms the suitability of this approach for MRKH patients.

To this aim we first demonstrated the presence of epithelial features, such as a cobble-stone-like shape, tight connections, and clear boundaries, of HVMs obtained from vaginal mucosal biopsy of MRKH patients. Our results were further confirmed by the detection through immunofluorescence analysis of the cytokeratin 19 (K19), one of the most common members of the intermediate filament proteins, specific for epithelial cells and responsible for their structural integrity [12].

Moreover, HVMs cultures established in our study showed high protein levels of the epithelial markers K19 and keratinocyte growth factor receptor (KGFR) through Western blot analysis. Conversely, the expression of vimentin, a mesenchymal marker, was not detected in these cells. The expression of epithelial markers (K5 and KGFR) and the lack of vimentin expression were also confirmed at mRNA level by means of qRT-PCR.

An interesting finding was the presence of mucified cells interspersed among cell cultures, as demonstrated by PAS staining. These cells secrete mucus, thus creating a protective supraepithelial layer acting as a barrier and mimicking the physiological action of the vaginal mucosa. Therefore, the use of HMVs layers for the canal lining might facilitate sexual intercourse and prevent infection through the spontaneous production of mucus.

Taken together, our data, showing expression of several epithelial markers such as K19, K5, and KGFR along with the presence of mucinous epithelial cells in HMVs population, strongly support the specific epithelial features of HMVs, necessary for an efficient epithelialization of the neovagina walls.

Finally, HMVs displayed an active growth capability and efficient expansion, characteristics necessary to obtain an adequate population of cells for autologous in vitro cultured vaginal tissue transplantation. Thus, epithelial HMVs offer a further advantage because large layers of cells can be cultured in about two weeks from 1 cm^2^ full-thickness mucosal biopsy obtained from the vaginal vestibule.

In light of these considerations, we believe that the autologous in vitro cultured vaginal tissue might represent the material of choice in modified McIndoe vaginoplasty since it has epithelial characteristics similar to the natural vaginal cells, the advantage of mucus production for neovagina lubrication and favorable growth performance in our culture conditions.

## 5. Conclusion

In conclusion, modified McIndoe vaginoplasty with autologous in vitro cultured vaginal tissue not only is a simple, minimally invasive and safe procedure but also creates a neovagina with an epithelial structure more similar to the physiological native vagina.

## Figures and Tables

**Table 1:** Characterization of MRKH patients.

Patient	Age (years)	Presentation	MRKH phenotype	Karyotype
1	18	Amenorrhoea	Type I	46, XX
2	30	Amenorrhoea	Type I	46, XX
3	32	Amenorrhoea	Type II	46, XX
4	17	Amenorrhoea	Type II	46, XX
5	18	Amenorrhoea	Type II	46, XX
6	28	Amenorrhoea	Type I	46, XX
7	16	Amenorrhoea	Type II	46, XX
8	23	Amenorrhoea	Type I	46, XX
9	18	Amenorrhoea	Type I	46, XX

**Table 2:** K5, KGFR, and vimentin gene expression in HVMs.

Ct	HVMs	MCF-7
**K5**	16,60 ± 0,19	15,773 ± 0,11
GAPDH	18,75 ± 0,13	18,21 ± 0,17

**KGFR**	27,81 ± 0,02	26,96 ± 0,01
GAPDH	19,08 ± 0,13	18,57 ± 0,06

**vimentin**	38,25 ± 0,01	36,25 ± 0,75
GAPDH	19,96 ± 0,016	17,72 ± 0,11

The mean and the standard deviation of the Ct values are based on triplicate analysis. GAPDH was used as endogenous control. MCF-7 served as an epithelial cell line control.
